# Self-Sensitization and Photo-Polymerization of Diacetylene Molecules Self-Assembled on a Hexagonal-Boron Nitride Nanosheet

**DOI:** 10.3390/polym10020206

**Published:** 2018-02-19

**Authors:** Elisseos Verveniotis, Yuji Okawa, Kenji Watanabe, Takashi Taniguchi, Takaaki Taniguchi, Minoru Osada, Christian Joachim, Masakazu Aono

**Affiliations:** 1 International Center for Materials Nanoarchitectonics (WPI-MANA), National Institute for Materials Science (NIMS), 1-1 Namiki, Tsukuba, Ibaraki 305-0044, Japan; TANIGUCHI.Takaaki@nims.go.jp (T.T.); OSADA.Minoru@nims.go.jp (M.O.); Christian.Joachim@cemes.fr (C.J.); AONO.Masakazu@nims.go.jp (M.A.); 2 Research Center for Functional Materials, National Institute for Materials Science (NIMS), 1-1 Namiki, Tsukuba, Ibaraki 305-0044, Japan; WATANABE.Kenji.AML@nims.go.jp (K.W.); TANIGUCHI.Takashi@nims.go.jp (T.T.); 3 Centre d’Elaboration de Matériaux et d’Études Structurales (CEMES), Centre National de la Recherche Scientifique (CNRS), 29 rue J. Marvig, 31055 Toulouse CEDEX, France

**Keywords:** polymerization, diacetylene, self-assembly, h-BN surface, molecular wires, AFM, self-sensitization

## Abstract

Long poly-diacetylene chains are excellent candidates for planar, on-surface synthesized molecular electronic wires. Since hexagonal-Boron Nitride (h-BN) was identified as the best available atomically flat insulator for the deposition of poly-diacetylene precursors, we demonstrate the polymerization patterns and rate on it under UV-light irradiation, with subsequent polymer identification by atomic force microscopy. The results on h-BN indicate self-sensitization which yields blocks comprised of several polymers, unlike on the well-studied graphite/diacetylene system, where the polymers are always isolated. In addition, the photo-polymerization proceeds at least 170 times faster on h-BN, where it also results in longer polymers. Both effects are explained by the h-BN bandgap, which is larger than the diacetylene electronic excitation energy, thus allowing the transfer of excess energy absorbed by polymerized wires to adjacent monomers, triggering their polymerization. This work sets the stage for conductance measurements of single molecular poly-diacetylene wires on h-BN.

## 1. Introduction

Current nano-device miniaturization is driving Si and Moore’s law to their limits. In search of materials for the electronics of the future, conjugated polymers [1,2,3] in general, and single electrically conductive molecular chains [4,5,6,7,8,9] in particular, have attracted considerable interest. Those chains, already utilized in transport measurements, will constitute the very core of molecular electronic circuits [8,9,10,11,12,13]. On-surface synthesis of such molecular chains was recently demonstrated in ultra-high vacuum-clean metal surfaces and at low temperature [14]. Diacetylene compounds (R–C≡C–C≡C–R′, where R and R′ are substituent groups) and their self-assembled monolayers (SAM) are therefore relevant for such applications because they on-surface synthesize to long isolated polydiacetylene (PDA) wires (=RC–C≡C–CR′=)*_n_* [8,15,16,17] in ambient conditions [8,18]. This surface topo-chemical chain reaction can be initiated by providing energy to the SAM for example via ultraviolet (UV) light irradiation, sample baking or electrical pulses using a scanning tunneling microscope (STM) tip. In addition to the on-surface reactions, polydiacetylenes are interesting for diverse applications such as optical [19] drug delivery and sensing [20].

Polymerized diacetylene chains are therefore excellent candidates for the on-surface fabrication of planar molecular electronic circuits. For this reason, the self-assembly of the PDA precursors must occur: (1) in a flat-lying manner to ensure device planarity; and (2) on an atomically flat insulator as the molecules are sensitive to surface corrugation [21] and for avoiding leakage currents or other unwanted electronic effects.

It was recently shown [22,23] that the hexagonal-Boron Nitride (h-BN) surface is a good candidate for this purpose, as it satisfies the two aforementioned conditions. However, due to its insulating nature, polymerization on the h-BN surface by an STM tip is not possible. We reported preliminary results that the UV-polymerization on h-BN is faster compared to the well-known diacetylene polymerization on highly oriented pyrolytic graphite (HOPG) [23]. Here, we evaluate the UV-polymerization rate more precisely and show that it is at least 170 times faster on h-BN than on a HOPG surface. In addition, the polymerization pattern on h-BN indicates PDA self-sensitization, effectively forming blocks of polymers instead of stand-alone chains. Both the self-sensitization and the enhanced polymerization rate occur due to the large electronic bandgap of h-BN. 

## 2. Materials and Methods

Nanosheets of h-BN typically tens of μm large were obtained by mechanical exfoliation from in-house synthesized ultrapure h-BN single crystals [24], using the adhesive tape method [25]. Substrates were 10 × 10 mm Si/SiO_2_ chips. After exfoliation we annealed the substrates at 500 °C for 2 h for adhesive tape residue and ambient adsorbate removal (h-BN is stable up to 1000 °C [26]). 

After substrate preparation, surface-deposition of the diacetylene molecules was performed by dropcasting a 4 μL droplet on the sample followed by spin-coating at 8000 rpm for 90 seconds. The diacetylene molecules used, 10,12-nonacosadiynoic acid (CH_3_(CH_2_)_15_C≡C–C≡C(CH_2_)_8_COOH), are amphiphilic with a hydrophilic COOH group at one end and a hydrophobic alkyl chain at the other end. The solution was prepared by adding 0.15 g/L of 10,12-nonacosadiynoic acid powder (Tokyo Chemical Industry Co., Ltd., Tokyo, Japan) in xylene. Note that this concentration has been optimized for the systematic formation of SAMs with minimal molecular debris accumulation elsewhere [22,23], and is appropriate regardless of the solvent being used [8,15,27,28]. Deposition on HOPG was done using the same method and solution on freshly cleaved samples (Structure Probe, Inc. West Chester, PA, USA).

Resulting structures were characterized by atomic force microscopy (AFM). Experiments were performed using a Keysight 5500 microscope (Keysight Co, Santa Rosa, CA, USA) and Multi75-G silicon cantilevers (Budget sensors, Sofia, Bulgaria) with 3 nm^−1^ spring constant and 75 kHz resonance frequency. Characterization was done in tapping mode due to the sensitivity of our materials. 

Photo-polymerization was performed by irradiating the samples under a low-pressure mercury UV lamp (λ = 254 nm, fluence = 1.3 mW/cm^2^). Exposure time varied from few seconds (h-BN) to several minutes (HOPG). Details on the UV-polymerization process of diacetylene can be seen in the literature [16,29,30,31]. AFM evaluation of the partially polymerized layers was performed by measuring more than three different places on each sample. The number and length of polymers were statistically calculated. Micro-Raman spectroscopy was carried out on a LabRAM HR Raman microscope with a laser excitation wavelength of 514 nm. Temperature and relative humidity during all experiments were in the ranges of 23−25 °C and 34−41%, respectively.

## 3. Results and Discussion

**Figure 1:**
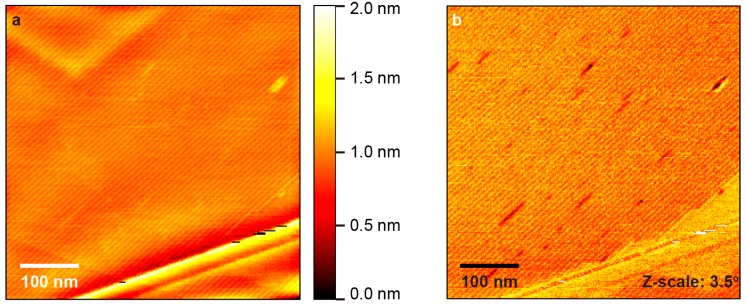
Atomic force microscopy (AFM) images showing diacetylene on highly oriented pyrolytic graphite (HOPG) after 20 min of ultraviolet (UV) irradiation: (**a**) topography; (**b**) phase shift.

Figure 1a shows the detailed AFM topography of a 500 × 500 nm^2^ area on HOPG with deposited 10,12-nonacosadiynoic acid after 20 min of irradiation. The long parallel stripes correspond to self-assembled molecular rows. In agreement with the literature [8], the stripe period is 7.5 nm. Several polymerized chains can be identified within the image as brighter from their surroundings. This is due to the lifted-up conformation of polymers with respect to their precursors [23,28]. Length of the polymers is under 100 nm, which is similar to the case of 10,12-pentacosadiynoic acid (CH_3_(CH_2_)_11_C≡C–C≡C(CH_2_)_8_COOH) on HOPG after 20 min [27] irradiation by the same UV lamp. This is expected, as differences in length and packing density between the two diacetylene compounds is within 10%. The polymers can be seen more clearly, as darker lines, in the corresponding phase shift image in Figure 1b.

**Figure 2:**
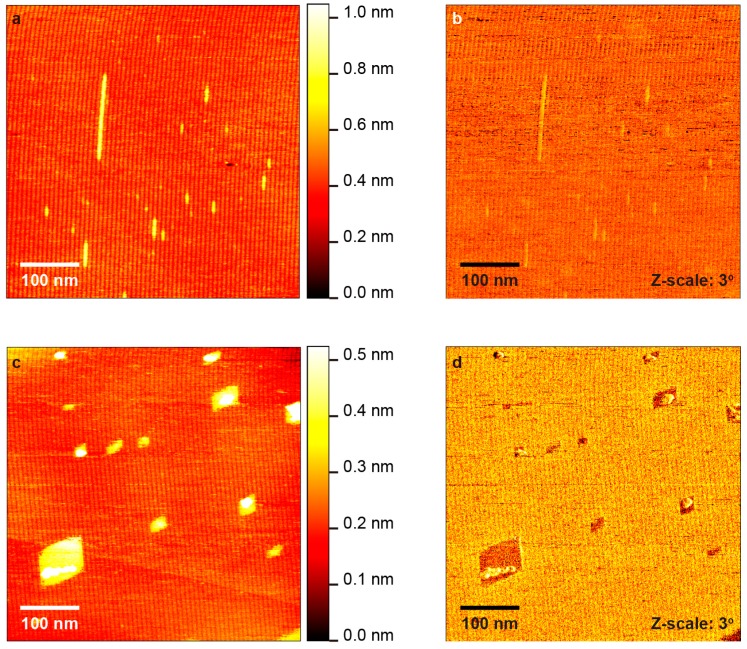
AFM topography (**a**); and phase shift (**b**) of diacetylene on hexagonal-Boron Nitride (h-BN) after 5 s of UV irradiation; and the same sample after additional 5 s of irradiation (10 s in total) (**c**) topography; (**d**) phase shift.

Figure 2a,b illustrates AFM topography (500 × 500 nm^2^) and corresponding phase shift, respectively, for diacetylene on h-BN after 5 s of irradiation. The stripe period is identical to the HOPG case. Several PDA chains with lengths under 100 nm can be identified. In addition, there is one polymer 160 nm long. Similar to the HOPG surface, the polymers appear higher than the surroundings. Polymerization rate on h-BN seems significantly faster as compared to HOPG. To confirm this point, we irradiated the same sample for five more seconds (i.e., a total of 10 s). AFM topography and phase shift are presented in Figure 2c,d. This is near the originally measured area in Figure 2a,b (within 10 μm) but clearly not the exact same spot. Due to the lack of impurities on the h-BN surface, it is nearly impossible to find the same area after further altering the topography by additional polymerization. We can see islands of polymerized material which confirm the enhanced polymerization rate on h-BN. Further irradiation for 1–2 min fully polymerizes the layer. Note that polymerized diacetylene exhibits a lifted-up structure which makes it stand 0.05–0.2 nm above the non-polymerized monomers, as evidenced by AFM [8,23,28]. In this lifted-up model, the innermost carbon atoms of the alkyl chains adjoining the PDA backbone, as well as the PDA backbone itself, are raised from the surface. The difference in the absolute height values is attributed to AFM probes and the measuring parameters (e.g., elastic deformation during the measurement). The height of islands in Figure 2c is measured to be ~0.20 nm. Therefore, changes in the AFM height within the SAM, in conjunction with corresponding phase shift changes, are a safe indication of polymerization occurrence. Nevertheless, one could critically call for further evidence such as chemical characterization. For this reason, we measured micro-Raman before and after UV-irradiation. 

**Figure 3:**
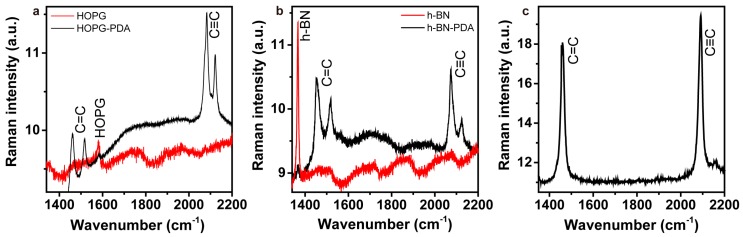
Raman spectra measured on: (**a**) pristine HOPG and HOPG after diacetylene deposition and UV irradiation for 20 min; (**b**) pristine h-BN and h-BN after diacetylene deposition and UV irradiation for 10 s; and (**c**) the diacetylene powder after UV irradiation for 10 s.

Figure 3a shows spectra measured on pristine HOPG, as well as after diacetylene deposition and 20 min of irradiation. The former spectrum shows only the HOPG G-band at 1580 cm^−1^ [32]. In the latter, we also see PDA-characteristic peaks. The bands at 1454 and 1514 cm^−1^ are attributed to the –C=C– stretching modes of the polymer backbone [33,34,35]. Similarly, bands at 2073 and 2123 cm^−1^ are due to –C≡C– backbone stretching variations [35,36,37]. In Figure 3b, we see spectra measured on pristine h-BN, and also after diacetylene deposition and UV-irradiation for 10 s. The same PDA-related bands show up here as well, together with the h-BN signature at 1368 cm^−1^ [38]. Note that providing energy to the system, in this case by the Raman laser, can induce polymerization of monomers within the investigated area. Therefore, the presented spectra correspond to polymerized material by both the UV-lamp and the Raman laser during spectrum acquisition. Nevertheless, the presence of polymers is confirmed. The same Raman peaks were also observed when measuring the pure diacetylene powder (deposited on a clean glass substrate) after irradiation for 10 s, as seen in Figure 3c.

After the second irradiation, PDA chains on h-BN appear next to each other, effectively forming islands of polymerized material. This is attributed to the well-known effect of self-sensitization, which causes partially polymerized crystals to further polymerize slightly faster than pure monomer crystals. The mechanisms proposed to date include reactivation of the chain end [39], homogeneous self-sensitization [40], and phonon excitation from neighboring rows [41]. However, after the second irradiation, we do not observe enhanced PDA chain lengths, the polymerization pattern is clearly inhomogeneous, and the initial irradiation produced individual polymers. Therefore, our work clearly indicates a different mechanism. 

**Figure 4:**
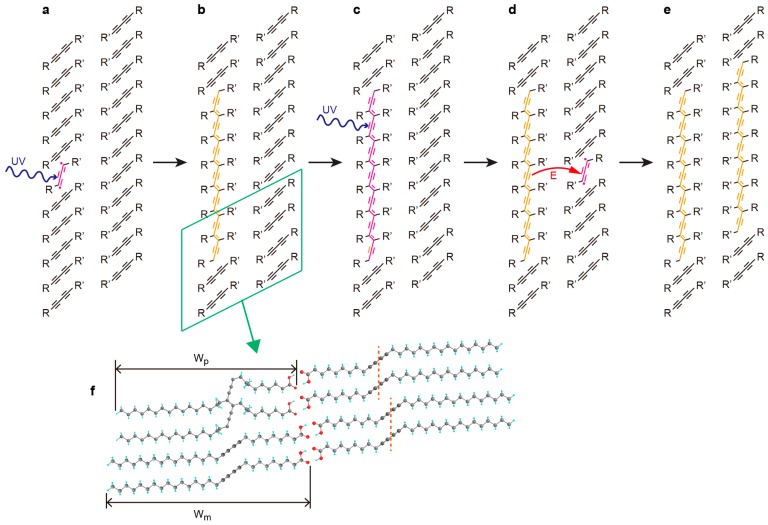
Schematic illustration of the self-sensitization of diacetylene on h-BN: (**a**) the diacetylene layer is exposed to UV-light and one of the molecules is excited; (**b**) a PDA chain is formed; (**c**) the PDA absorbs energy due to further irradiation; (**d**) energy is transferred to the neighboring monomer; and (**e**) this causes it to polymerize. R and R’ are (CH_2_)_15_CH_3_ and (CH_2_)_8_COOH, respectively. (**f**) Detailed model of the area denoted by the rectangle in **(b)**. Orange dotted lines show the monomer arrangement defect near the polymer edge. *W*_p_ and *W*_m_ indicate the width of polymer and monomer, respectively.

Here, we propose a mechanism as shown in Figure 4, where the initial polymerized chains adsorb light, providing the energy to neighboring monomers. First, UV light is being shone on the SAM of diacetylene (Figure 4a). This results in the formation of a PDA chain as seen in Figure 4b. Continuing to irradiate the same area allows the absorption of energy by the polymer (Figure 4c). The energy is then transferred to the nearby monomer causing it to polymerize as seen in Figure 4d,e. The wide bandgap of h-BN (5.97 eV) [42] can elongate the excited polymer state lifetime (Figure 4c) and thus clearly enhance the probability of this process. In contrast, on HOPG, the excited polymer energy will rapidly relax into the substrate due to the lack of bandgap, thus not allowing the formation of the second polymer. Another possibility for the observed effect can be the enhanced reactivity of PDA-neighboring molecular rows. This is caused by the lifted-up structure of the PDA chains [28] that are narrower (width shown as *W*_p_ in Figure 4f) than the monomer rows (width shown as *W_m_* in Figure 4f), causing increased neighboring monomer mobility. The less adsorption enthalpy of molecules on h-BN compared to HOPG [43] can also affect the probability of this process.

In Figure 2c, we can also see that the length of polymers per 2D self-sensitized PDA island is almost the same. This is due to the polymer width which is smaller than that of monomer rows as already discussed, which induces defects of monomer molecular arrangement near the terminal of a neighboring polymer (Figure 4f). The second polymerization will be thus terminated at that defect. As a result, the length of the second polymer will be the same as that of the first polymer.

**Figure 5:**
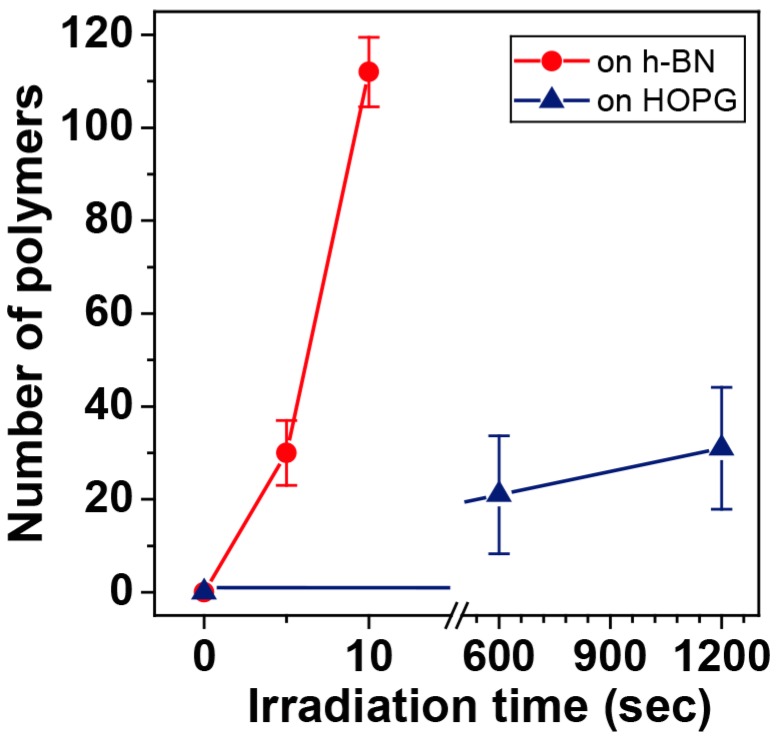
Number of polymers in a 500 × 500 nm^2^ area as a function of irradiation time for the photo-polymerization of 10,12-nonacosadiynoic acid on HOPG and h-BN. The lines are guides for the eye.

The reason for the enhanced photo-polymerization rate of diacetylene on h-BN vs. HOPG is also attributed to the large electronic bandgap of the former. Since the excitation energy of diacetylene is about 3 eV, it can relax on the HOPG substrate as graphite has no bandgap. This is corroborated by photo-polymerization on MoS_2_, as its bandgap (1.2 eV) is smaller than the diacetylene excited state energy which also allows it to relax results in a similar photo-polymerization rate as the HOPG system. However, the large bandgap of h-BN suppresses the relaxation mechanism, leading to more on-surface reactions that are translated to more polymers on the surface of h-BN. Polymer count as a function of time, on both substrates, and averaged from several experiments, is summarized in Figure 5. Based on those results photo-polymerization rate on h-BN is at least 170 times faster than on HOPG. Note that the difference in the polymerization rate is a function of irradiation time, as in the case of h-BN self-sensitization causes a rapid increase in the reactivity which inherently increases the rate with exposure time. Therefore, quantifying the polymerization rate by a single value would not be correct.

Besides the polymer count after irradiation, we also observed a significant difference in the averaged polymer length between substrates. On HOPG, the averaged polymer length is slightly increasing or stays almost constant within the error: 28 ± 7 nm for 10 min and 42 ± 16 nm for 20 min irradiation, corresponding to 60 and 90 monomers per polymer chain, respectively (distance between monomers is 0.47 nm [8]). This indicates that each polymerization on HOPG is an individual event. On the other hand, the averaged length increases significantly on h-BN, where we count 23 ± 3 nm for 5 s and 116 ± 76 nm for 10 s irradiation (50 and 250 monomers per PDA chain, respectively). For h-BN, statistically, the amount of energy (photons) absorbed by a polymer, and potentially transferred to its nearby monomer, should be proportional to its length. Thus, a longer polymer absorbs more the light and as a result induces the second nearby polymerization more frequently compared to a shorter polymer. Such effect is partially responsible for the self-sensitization observed in the h-BN/PDA system as seen in Figure 2c, where the islands of longer polymers are comprised by more polymers as well. In addition, the length of polymers per 2D self-sensitized PDA island is almost the same as already discussed. From the above we can conclude that the formation of long polymers should be faster when compared to shorter ones, which explains the difference in the length after irradiation on h-BN. The fact that the longer polymers show enhanced self-sensitization also supports our proposed mechanism, where the photo-absorption of the first polymer induces the following reactions.

## 4. Conclusions

In this work, we show that the 10,12-nonacosadiynoic acid molecules surface-polymerize on HOPG in a similar fashion as the 10,12-pentacosadiynoic acid when irradiated under a UV lamp. However, photo-polymerization of diacetylene compounds deposited on h-BN forms islands of polymerized material due to self-sensitization. In addition, the polymerization rate is demonstrated to be at least 170 times faster on h-BN vs. on HOPG, effectively leading to more and longer polymers after only few seconds of irradiation. Both effects are attributed to the large electronic bandgap of h-BN. These results should be considered in future work towards PDA-based molecular electronic circuits, as they demonstrate that an insulator with a bandgap significantly larger than the excitation energy of diacetylene (~3 eV) is necessary for efficient layer polymerization. As a next step, the stage is ready for the fabrication of simple source-drain devices based on h-BN and PDA for conductance measurements, initially via self-sensitized polymer islands, and finally through a single PDA chain.
